# Lectures on Genito-Urinary Diseases

**Published:** 1894-09

**Authors:** 


*Lectures on Genito-Urinary Diseases.* By J. C. Ogilvie Will, M.D. Pp. 152. London: The Scientific Press, Limited. 1894.

This book, consisting of six lectures delivered in the Aberdeen Royal Infirmary, deals in a readable and practical manner with the more common varieties of genito-urinary diseases, and has some useful illustrations. The author states that the views expressed in the chapter on urethral fever and catheter fever received the approval of the late Sir Andrew Clark—who took much interest in the subject of fever following urethral instrumentation—and were written partly at his request. The details of treatment of the several diseases are in most cases fully described, and while the book can be specially recommended to the student and young practitioner, it will serve to recall various details of diagnosis and treatment to those more experienced.

